# Identifiable regional and neural network abnormalities in early childhood anxiety

**DOI:** 10.1002/jcv2.70158

**Published:** 2026-08-16

**Authors:** Khalil I. Thompson, Yiwen Zhang, Denny Schaedig, Anna Bankston, Anoushka Parnerkar, Shrinidhi Jeyaram, Nadine Melhem, Susan B. Perlman

**Affiliations:** ^1^ Department of Psychiatry School of Medicine Washington University St. Louis Missouri USA; ^2^ Department of Psychiatry School of Medicine University of Pittsburgh Pittsburgh Pennsylvania USA

**Keywords:** anticipatory fear, anxiety, early childhood, fMRI, free‐viewing, ICA

## Abstract

**Background:**

Although the neural underpinnings of anxiety have been investigated, most research has been confined to adolescence and adulthood, with less of a focus on the early childhood period. Little is known about the role of anticipatory fear processing early childhood, which is a sensitive period for anxiety development. Free‐viewing paradigms provide a useful method for investigating brain activation during fear anticipation because they present stimuli in a manner that mirrors the participant's natural environment.

**Methods:**

One hundred and fifty‐nine, high‐risk children (*M* = 5.93 years; SD = 1.16) (54% female) watched an episode of a children's animated program that relied on anticipatory fear for the main plot line. Parents rated children on anxiety symptoms. Data was analyzed using both general linear modeling (GLM) and independent components analysis (ICA) to determine task‐dependent regional and network response to positively and negatively‐valenced time‐series in the episode.

**Results:**

GLM analyses identified significant positive regional activation in the dorsomedial prefrontal cortex, middle temporal gyrus, and thalamus that fluctuated during the negative timecourse as a function of anxiety symptoms. Furthermore, ICA analyses revealed diminished network connectivity between (1) the anterior cingulate and raphe nuclei and (2) the right temporoparietal junction, left superior parietal lobule, and lingual gyri as a function of anxiety symptoms.

**Conclusion:**

Our findings point to fractured communication between emotion and sociocognitive processing regions as a function of increased anxiety in early childhood. The findings of this study can point to neural mechanisms present in early anxiety symptomatology before the traditionally defined onset.

## Key Points

**What's Known?**

- Anxiety symptoms can emerge as early as preschool, but neural mechanisms in this age group are understudied compared to adolescents and adults.

**What's New?**

- This study used naturalistic fMRI (a children's TV show) to identify brain regions (dorsomedial prefrontal cortex, middle temporal gyrus, thalamus) associated with anticipatory fear in 4–7‐year‐olds with higher anxiety.
- Altered connectivity and power spectra in the anterior cingulate, raphe nuclei, and temporoparietal junction suggest early disruptions in emotion regulation and social processing networks.

**What's Relevant?**

- This study supports early screening for anxiety using ecologically valid tools and offer a foundation for developmentally tailored interventions.
- Longitudinal follow‐up is essential to confirm whether early neural markers predict later anxiety disorders, and to identify critical windows for intervention.

## INTRODUCTION

Anxiety disorders are amongst the most prevalent mental health conditions in children and adolescents (Ollendick & March, 2004). However, researchers have generally focused on anxiety and its biomarkers in adolescence and adulthood, with less focus on the neural substrates of anxiety symptoms during early childhood. Indeed, a recent model suggests the presence of “sensitive periods” for the expression of psychopathology, characterized by disorder‐dependent alterations in the development of age‐appropriate behavior and underlying neural circuitry (Uhlhaas et al., 2023). This theory highlights the age of 5.5 as the peak of heightened risk for the emergence of anxious symptomology, a time point which significantly precedes the average age of onset of other disorders (age 12–25) (Solmi et al., 2022). Thus, an important research direction is to understand the biological mechanisms that may precipitate and underlie anxiety symptoms while also clarifying why anxiety symptoms might manifest at such early ages.

Anxiety is often conceptualized as a future‐oriented state characterized by anticipatory responses to uncertainty or the possibility that a potential threat or aversive outcome may occur (Carleton, 2016; Grupe & Nitschke, 2013). Prior work has shown that preadolescent children aged 8–12 years with anxiety exhibit exaggerated anticipatory fear responses (Williams et al., 2015). However, the role of anticipatory fear in the emergence of anxiety symptoms in younger children remains poorly understood (Warren & Dadson, 2001). In early childhood, anxiety‐related processes are often assessed through observable fear, avoidance, behavioral inhibition, or attentional bias toward threat, whereas future‐oriented anticipatory processes are more difficult to measure directly (Mian et al., 2015; Spence et al., 2001). This likely reflects, in part, ongoing development of future‐oriented cognition; for example, episodic future thinking remains immature in 4‐year‐old children (Cuevas et al., 2015). As a result, anticipatory fear may be particularly difficult to capture in young children, who have limited introspective and verbal abilities. Consistent with this view, behavioral studies show that 4‐ to 5‐year‐old children can acquire fear through verbal threat information, which subsequently manifests as increased avoidance rather than explicit anticipatory report (Field & Lawson, 2003; Rifkin et al., 2016). Despite these measurement challenges, available evidence suggests that children with elevated trait anxiety or anxiety symptoms show heightened conditioned anticipatory fear responses to unpredictable threat (Field & Storksen‐Coulson, 2007; Lester et al., 2011; Nelson & Hajcak, 2017). While existing work in young children has largely emphasized behavioral outcomes, developmental neuroimaging studies in older children and adolescents provide an important foundation for identifying the neural mechanisms through which anticipatory fear may contribute to emerging anxiety symptoms.

The developmental literature investigating the neural substrates of anxiety symptoms and anticipatory fear has primarily targeted preadolescent and adolescent children. Voxelwise activation studies demonstrated that adolescents with both an anxiety disorder and heightened intolerance of uncertainty exhibited an elevated neural response in the anterior cingulate (ACC), orbitofrontal cortex and left amygdala commensurate with the likelihood of an incorrect response (Kra et al., 2008). Further, one recent project indicated that preadolescent youth with anxiety disorders displayed heightened neural response in the ventrolateral prefrontal cortex (vlPFC) and reduced response in the ventral striatum during uncertain threat anticipation (Michalska et al., 2023). Subsequent research employing functional connectivity approaches demonstrated that preadolescent children with anxiety disorders experience differential amygdala activation and functional connectivity when anticipating the presentation of fearful or neutral faces (Feola et al., 2021; Williams et al., 2015). In a middle childhood sample, network‐level analyses revealed that anxious children exhibited reduced connectivity between these prefrontal regions, and the amygdala, and increased connectivity between central executive functioning and salience network faculties (Clauss et al., 2016). Taken together, voxelwise activation studies have consistently implicated focal regions including the amygdala, ACC, and vlPFC, whereas connectivity‐based approaches have revealed broader network‐level dysregulation within frontostriatal and salience circuits. These complementary findings suggest that a comprehensive characterization of anxiety‐related anticipatory processing requires both methodological approaches. While researchers have made significant progress in characterizing the neurobiological mechanisms of anticipatory fear and tolerance of uncertainty in older youth, there is a scarcity of literature investigating this question in early childhood (ages 4–7).

Task paradigms that are typically administered to study anticipatory fear in children often either lack the social content that defines the rich environment of a developing child (e.g., simple card games), or they leverage static stimuli that do not effectively simulate the dynamic nature of social interaction (e.g., static emotional faces). Importantly, empirical evidence suggests that vicarious threat anticipation, such as observing others in danger, recruits the same neural circuitry (e.g., amygdala, ACC) as direct personal threat experience (Olsson & Phelps, 2007). Narrative films with suspenseful content are particularly effective at engaging these neural circuits (Kirk et al., 2023), supporting the validity of film‐based paradigms for studying anticipatory threat processes. Furthermore, free‐viewing paradigms serve as an excellent tool for exploring underlying brain function during sensitive developmental periods because they present daily life stimuli in a realistic manner that mirrors the participant's natural environment or general media viewing habits. Free‐viewing paradigms allow participants to engage with stimuli in a spontaneous and flexible manner, increasing the ecological validity and real world applicability of the task—a challenge often faced in neuroimaging studies (Camacho et al., 2019; Karim & Perlman, 2017). Furthermore, they may be more robust than traditional paradigms when testing clinical populations and children under 10 years (Vanderwal et al., 2019).

The current study investigated the neural correlates of anxiety symptoms and the anticipation of negative outcomes in young children using fMRI. Informed by the previous findings using voxelwise and connectivity methods in older youth, we employed a dual‐methodological approach by using traditional general linear modeling (GLM) to identify specific regional activation responses (Friston et al., 1994), and independent components analysis (ICA) to characterize large‐scale functional network connectivity (FNC) architecture in a data‐driven manner (Calhoun et al., 2001). Based on this integration of previous voxelwise and connectivity research, we hypothesized that heightened trait anxiety in preschool‐age children would be associated with both increased localized activation and altered FNC in regions comprising the frontostriatal (PFC and striatum) and fear conditioning/salience (amygdala, ACC, and insula) networks when exposed to fluctuating, negatively‐valenced stimuli.

## METHODS AND MATERIALS

### Ethical considerations (IRB approval)

This study was approved by the Institutional Review Board (IRB) at Washington University in St. Louis. All parents provided written consent and children provided verbal assent. Data was collected as part of the Child Affect and Resilience to Experiences study, originally designed to investigate the biological unfolding of the stress system as a result of interparental conflict.

### Sample characteristics

We enrolled 229 preschool‐aged children (ages 4–7) from the local community. Although it is not the focus of the current project, the sample was enriched for increased interparental conflict, which is a risk factor for early and lasting psychopathology (Harold & Sellers, 2018). Children were recruited from the community, but also using recent county court divorce records and recruitment from clinics specializing in parental separation. Participants were also high in adverse childhood experiences (ACEs; Felitti et al., 1998) aside from interparental conflict. While 31% had no ACEs, the remaining subjects had at least one ACE (1 ACE = 30%; 2 ACEs = 12%, 3 ACEs = 8%; 4 or more ACEs = 19%). Participants were recruited as part of a three visit 18‐month longitudinal study. The fMRI data reported here were collected at the baseline timepoint of the study (V0). All 229 children were invited to participate in the MRI scan, however 22 chose not to participate or did not show up for the scan visit, leaving a sample of 207 potential subjects. Of the 207, 11 were too fearful to attempt the scan, 2 did not pass the MR safety screening, and 2 exhibited non‐compliant behavior that made MRI unsafe, leaving 192 children that entered the scanner environment. Among those 192 children, 19 terminated the scan before the free‐viewing task began, 12 did not produce sufficient data for analysis (i.e., terminated the scan mid‐task), and 2 had file corruption, making their data unusable. Therefore, 159 children who met the head motion quality control criteria (mean FD < 0.5 mm) comprised the final participant sample, and volumes exceeding this threshold were censored. This procedure yielded high data retention rates for Movie A (81.8%), Movie B (88.7%), and Movie C (87.7%), corresponding to an average of 907, 1099, and 1075 s of usable data, respectively.

Among the 159 children who completed scanning, the average age was 5.93 years (SD = 1.16) (54% female). The children were identified by their parent as African‐American (11%), Asian (1%), multiracial (19%) and White (69%). Ten percent identified as Hispanic. Annual household income distribution was as follows: less than $20,000–$39,999 (6.3%), $40,000–$59,999 (14%), $60,000–$99,999 (24%), $100,000–$119,000 (11%), $120,000–$250,000 (31%), more than $250,000 (12%).

### Questionnaire measures of anxiety symptoms

The MacArthur Health and Behavior Questionnaire (HBQ) is a widely‐used, parent‐report measure assessing children's emotional, physical, behavioral, and social health (Boyce et al., 2002). The HBQ parent‐report form includes a broad set of items assessing multiple domains of child functioning, with specific symptom subscales derived from subsets of items. We focused our analyses on the Overanxious subscale, which consists of 12 items assessing children's anxiety and worry symptoms. Items include “worries about things in the future,” “needs to be told over and over that things are ok,” and “nervous, high strung, or tense.” All Overanxious items are scored on a 3‐point scale (0 = Never or not true, 1 = Sometimes or somewhat true, 2 = Often or very true). The final subscale score is calculated as the mean of these 12 items, resulting in a theoretical possible range of 0.0–2.0. The HBQ has demonstrated reliability and validity for assessing emotional and behavioral symptoms in child samples, including internalizing symptoms in children under 9 years of age. Thus, the Overanxious subscale was used as a dimensional measure of individual differences in parent‐reported anxiety and worry symptoms, rather than as a diagnostic measure of clinical anxiety disorder. In our sample, the mean HBQ Overanxious score was 0.32 (SD = 0.27), with an observed range of 0.00–1.25. Cronbach's *α* was .77, 95% CI [0.72, 0.82], indicating acceptable internal consistency (Taber, 2018).

### Task description/procedure

Participants engaged in a free‐viewing fMRI task in which they watched an episode of the children's animated television show “All Hail King Julien” (Benear et al., 2023). This series was chosen because (1) the show has a quick moving plot that repeatedly jumps from high to low emotional states, building anticipation and generating uncertainty about the events of the episode and (2) the animal protagonists of the series avoid the need to match actors to the diverse races and ethnicities of our subjects. During scanning, an experimenter was present in the scanner room to help children manage any distress, and to monitor that participants kept their eyes open and attended to the film. Due to the longitudinal nature of our study, three different episodes from the series were chosen for the subject's three longitudinal visits, with the order in which the subjects viewed the episodes randomized across visits. Data is presented from the initial visit (V0). Final usable data included 53 subjects viewing episode A, 51 viewing episode B, and 55 viewing episode C. The plots of each story line heavily rely on generating anticipatory fear with the story's protagonist (a lemur) unaware of the impending danger (predators) who are often sneaking up behind him and viewable only to the audience. Episode (run) length ranged from 21.4 to 22.7 min. To be included for analysis, participants had to have watched at least 40% of an episode (>8.5 min), which exceeds the 5–7 min required to yield stable functional connectivity estimates, particularly in pediatric naturalistic viewing (Birn et al., 2013; Fox et al., 2005; van der Meer et al., 2020; Van Dijk et al., 2010). Furthermore, continuous emotion coding confirmed that negatively‐valenced content was distributed throughout each episode rather than temporally concentrated. Importantly, total usable viewing duration was not significantly correlated with HBQ Overanxious scores (*r* = .10, *p* = .225), indicating no systematic bias from data retention.

### Dynamic emotion coding

To characterize the dynamic changes in emotion content of the task stimuli, we followed measures outlined by Pantelis et al. (2015). Ten individual coders independently watched the stimulus movies while online coding emotional content in real time. Using a joystick, coders pushed the stick up as emotional content became more positive (score of 0.01–1.0) and down as content became more negative (score of −0.01 to −1.0), keeping the joystick in the middle position when emotion was neutral (score of 0). Coders were instructed to consider the affective content within the generally mild context of a children's TV show, to use the full range of emotion in their coding, and to keep the joystick in constant motion. Coding produced a time‐course of emotional scores, which was then down‐sampled to the rate of the scanner TR (0.8 s) and averaged at every timepoint across all 10 coders to create an average time‐course.

The intraclass correlation (ICC) at each timepoint ranged from 0.40 to 0.85 with an average ICC of 0.71, indicating good agreement of emotional content between coders. These dynamic shifts in emotional content, which we refer to as “fluctuations” throughout the manuscript, capture both the temporal variations in movie valence and the corresponding moment‐to‐moment changes in blood oxygen‐level dependent (BOLD) signal. The three episodes encompassed diverse emotional profiles: Movie A was balanced (47.9% positive vs. 52.1% negative), Movie B was positive‐skewed (60.2% vs. 39.8%), and Movie C was negative‐skewed (24.3% vs. 75.7%). This heterogeneity is an intrinsic characteristic of naturalistic viewing paradigms, which prioritize ecological validity over the strict experimental control typical of conventional block‐design fMRI tasks. Episode assignment was randomized across participants, and HBQ Overanxious scores did not differ significantly across the three episode groups (*F* = 0.95, *p* = .388). Moreover, because BOLD responses were modeled parametrically using the continuous emotion time‐series from each participant's assigned episode, neural estimates reflected responses to the emotional fluctuations each child viewed rather than episode‐level valence categories. This approach minimized potential confounds related to between‐episode variability in emotional skewness. Although continuous emotion ratings were obtained from adult coders, this approach was adopted because continuous emotion coding proved infeasible for our youngest participants (as young as age 4), as children tended to respond in extremes on the rating scale without intermediate variation (Chambers & Johnston, 2002), became distracted and stopped rating, or lacked the motor‐cognitive coordination required to perform fine joystick movements while sustaining attention to emotional content over extended periods.

### MRI data acquisition

Neuroimaging data were collected using a 3.0 T Siemens Magnetom Prisma MRI Scanner (Siemens). The scanner was equipped with a 10‐channel head coil used to acquire high‐resolution structural T1 contrast images using a magnetization prepared rapid gradient echo pulse sequence at the start of each session (TR = 2300 ms, TE = 3.14 ms, flip angle = 7°, FOV = 213 × 208 × 213.0 mm, anisotropic 0.83 × 1 × 0.83 mm voxel, 240 LR × 256 PA × 208 IS, total acquisition time = 111.30 s, 1 volume). Functional whole brain BOLD multi‐echo planar images were collected in a sagittal acquisition (TR = 800 ms, TE = 64.32 ms, flip angle = 49°, FOV = 204 mm, 3 mm isotropic voxels, 68 LR × 68 PA × 40 IS). The specific acquisition durations for Movie A, Movie B, and Movie C were 1283.2 s (1604 volumes), 1363.2 s (1704 volumes), and 1320.8 s (1651 volumes), respectively.

### MRI preprocessing

To preprocess fMRI data, we utilized the fMRIprep pipeline (version 23.2.0) (Esteban et al., 2019), which performs robust standard preprocessing steps including: brain extraction, spatial normalization, motion correction, fieldmap‐based distortion correction, slice‐timing correction, co‐registration to the T1‐weighted structural image, and confounds estimation (motion and sensory confounds). MRI data from all subjects were visually inspected for excessive motion and to ensure that BOLD scans were aligned with the T1w anatomical image and Montreal Neurological Institute (MNI) space. All masks generated for brain, white matter and cerebrospinal fluid (CSF) extraction were checked for adequate coverage of pertinent regions. Rotational and translational motion regressors alongside framewise displacement were produced through MCFLIRT. Motion was corrected during fMRIprep through spatial realignment using the motion regressors within during the interpolation step to minimize blurring. Loudness and luminance regressor's were produced using a custom python program to assess the average loudness and luminance of the movies for each volume. Cortical and sub‐cortical segmentation was done using Recon‐all. White matter, gray matter and CSF segments are determined using FSL FAST. BOLD scans are registered to a standard volume space to extract motion confounds during MCFLIRT. Through non‐linear registration via ANTs, BOLD scans are warped to standard templates such as MNI152NLin2009cAsym. BOLD scans are registered to anatomical scans through boundary‐based registration. Data was normalized to the standard MNI space using the MNIPediatricAsym (cohort 2) output space with a 2 mm isotropic resolution. Intensity correction was applied to improve boundary‐based registration by using N4 bias field corrections and intensity normalization against a reference volume. This reference volume was the mean of selected volumes. Physiological noise was accounted for through CSF and white matter regressors included in lower‐level analysis. The 7 mm kernel was selected to align with the native 3 mm acquisition resolution, ensuring compliance with the smoothness assumptions of random field theory for the subsequent GLM analysis implemented in Statistical Parametric Mapping (SPM), and to improve the whole‐brain signal‐to‐noise ratio. Post fMRIprep preprocessing, BOLD data was smoothed with a 7 mm kernel using FSL and motion and CSF confounds extracted from fMRIprep derivatives. Anatomical T1‐weighted (T1w) images were preprocessed by alignment to a common space with right‐anterior‐superior orientation and a uniform voxel size, later to be used as a basis for subsequent skull‐stripping, segmentation, and spatial normalization. The brain is then extracted using the antsBrainExtraction tool from ANTs. FSL's FAST is then used to segment the brain tissue into gray matter, white matter, and CSF. The anatomical image is then normalized to a standard space using ANTs' antsRegistration, which performs nonlinear registration with a mutual‐information based cost function.

### Data Analysis

In present study, fMRI data were analyzed using both univariate GLM and ICA. For the GLM approach, we implemented model design, estimation, and analysis in SPM12 to calculate event‐related average BOLD response amplitudes at the individual subject and group levels. Two primary regressors (representing dynamic variability in positively and negatively valenced content) were structured and entered as time‐series regressors. Six translational and rotational motion regressors and two sensory noise regressors (loudness and luminance) were included in the design to provide further control of head motion‐related variance and variance associated with auditory and visual stimuli not directly related to the emotional scenes of interest for the study. After the first‐level models were specified and estimated, the following subject‐level contrasts were generated: (a) variability in negative content, (b) variability in positive content, (c) negative > positive, and (d) positive > negative. To examine associations between anxiety symptoms and task‐evoked BOLD responses, HBQ Overanxious scores were then entered as a continuous predictor in second‐level regression models for each subject‐level contrast map. For all GLM analyses, statistical maps were thresholded using an uncorrected voxel‐wise threshold of *p* < .001 and a cluster‐level false discovery rate (FDR) corrected threshold of *p* < .05 (Verhoeven et al., 2005).

For the ICA approach, group spatial ICA was performed using GIFT (Group ICA fMRI Toolbox, http://icatb.sourceforge.net/). The default GIFT mask procedure was used, which creates a group mask from the mean functional image across all subjects, restricting ICA decomposition to brain tissue and excluding non‐brain voxels. Infomax was chosen as the ICA algorithm with default parameters (subject‐specific group PCA, GICA back reconstruction, mean signal removed per timepoint for preprocessing, etc.). The original number of ICs (78) estimated by the minimum description length approach yielded a strong number of reliable ICs based on cluster stability. Task‐related ICs were identified using the temporal sorting procedure implemented in GIFT (Calhoun et al., 2008; Malinen & Hari, 2011). Specifically, component time courses were regressed against the SPM design regressors corresponding to positive and negative movie‐content fluctuations. The resulting model fit was summarized using *R* ^2^, reflecting the proportion of variance in component time courses explained by the task regressors. Components significantly associated with the task regressors were identified using one‐sample *t*‐tests at *p* < .001, uncorrected. To characterize whether retained task‐related components were preferentially associated with positive or negative movie‐content fluctuations, component associations with the positive and negative regressors were compared using one‐way ANOVAs. Components identified as motion‐related or non‐neural artifacts based on their spatial maps and temporal profiles were excluded from subsequent analyses. The remaining task‐related ICs were retained for subsequence analyses.

Following previous work, functional network interactions between the ICs of interest and covariation with our HBQ Overanxious score was computed using the MANCOVAN toolbox in GIFT (Allen et al., 2011). MANCOVAN utilizes a general univariate model to estimate the latent “features” of a preexisting ICA dataset, including (a) component spatial maps, (b) component power spectra, and (c) FNC. Spatial maps reflect the spatial distribution and coactivation pattern of voxels within each component. Power spectra refer to the neural signal strength and coherence at specific frequencies within a component. In the neurotypical brain, functional coherence in signal oscillations is primarily found in the lower frequency bands (0.01–0.15 Hz) (Cordes et al., 2000; Sun et al., 2004). Finally, FNC refers to Pearson's correlations between time courses of components. Fisher's *Z* transformation was applied, and a symmetric matrix of FNC values was produced. Finally, the psychometric variables were included as covariates of interest in relation to the features produced by the MANCOVAN algorithm. Multiple comparisons were controlled using FDR correction (*p* < .05) as implemented by the *mafdr* function in GIFT.

Because the parent study sample was enriched for ACE exposure, particularly interparental conflict, both of which are closely associated with childhood anxiety symptoms (Elmore & Crouch, 2020; Ran et al., 2021), ACE burden may influence the interpretation of anxiety‐related neural findings. To examine whether the primary results remained robust after accounting for this broader adversity‐related influence, we repeated all MANCOVAN analyses with total ACE score included as an additional covariate in a supplementary sensitivity analysis.

## RESULTS

### GLM analysis

#### Time‐series contrasts

During fluctuations across the task time‐series associated with positively‐valenced movie content, significant task‐related BOLD activity was detected in clusters spanning multiple subregions of the visual cortex and the posterior cingulate. During time series fluctuations associated with negatively‐valenced content, we observed significantly elevated BOLD activation in the left superior temporal gyrus (STG) and the right rolandic operculum (Table 1).

**TABLE 1:** Significant clusters of activation in response to fluctuating emotional content of free‐viewing task.

Name of region	Brodmann area	Voxel	MNI coordinates			*T* value	*p‐*value (*p* < .05; FDR)
			*X*	*Y*	*Z*		
Positive							
Visual cortex	17	20,102	16	−88	−2	9.20	.001
Posterior cingulate	7	363	0	−62	30	3.95	.001
Negative							
R middle temporal gyrus	20	351	70	−20	−10	3.78	.001
Pos > neg							
Visual cortex	17	12,831	44	−70	2	6.56	.001
Neg > pos							
L middle temporal gyrus	21	282	−68	−24	−14	4.12	.004
R middle temporal gyrus	20	423	66	−22	−12	4.09	.001
R inferior parietal lobule	40	391	54	−46	44	4.05	.001
R middle frontal gyrus	9	222	36	42	32	3.55	.008
Posterior midcingulate	24	182	4	−26	48	3.44	.015
Dorsal ACC	32	121	2	34	20	3.41	.041

*Note*: Minimum *t*‐stat threshold = 3.14. All results obtained using an uncorrected voxel‐wise threshold of *p* < .001 and a FDR cluster‐corrected threshold of *p* < .05.

Abbreviations: ACC, anterior cingulate; FDR, false discovery rate; MNI, Montreal Neurological Institute.

#### Direct contrasts

When directly contrasting the positive and negative movie time‐series, fluctuations linked to positively‐valenced time series evoked significantly greater BOLD activation in the visual cortex compared to negative‐valenced time‐series fluctuations. However, the negatively‐valenced time‐series evoked greater activation than the positively‐valenced time series in the dorsal anterior cingulate cortex, right posterior insula, left anterior insula, bilateral inferior parietal lobules, right middle temporal gyrus (MTG) and left dorsolateral prefrontal cortex (dlPFC).

### GLM analysis (anxiety symptom correlates)

#### Time‐series contrasts

In the positive‐fluctuation contrast, no significant positive associations were observed between the HBO Overanxious scores and BOLD responses. However, we did detect a significant negative association between the Overanxious subscale and BOLD response to fluctuations in the positively‐valenced time series in the right STG (*t* = 3.55, *p* = .044) and right inferior frontal gyrus (*t* = 3.51, *p* = .030). During negatively‐valenced time‐series fluctuations, we detected a significant positive association in the dorsomedial prefrontal cortex (dmPFC; *t* = 3.56, *p* = .014), while no significant negative associations were identified (Table 2).

**TABLE 2:** Significant clusters of activation in response to fluctuating emotional content that correlate with the Overanxious subscale of the HBQ.

Name of region	Brodmann area	Voxel	MNI coordinates			*T* value	*p‐*value (*p* < .05; FDR)
			*X*	*Y*	*Z*		
Positive (Act)							
N/A							
Positive (Deact)							
R superior temporal gyrus	22	211	50	−22	−2	3.55	.044
R inferior frontal gyrus	9	253	42	16	22	3.51	.030
Negative (Act)							
dmPFC	10	263	2	66	18	3.56	.014
Negative (Deact)							
N/A							
Neg > pos (Act)							
dmPFC	10	2994	2	64	18	4.26	.001
R middle temporal gyrus	21	428	54	4	−26	3.66	.001
L thalamus		275	−14	−14	2	3.42	.013
L hippocampus		179	−24	−20	−18	3.77	.057 (trend)
Neg > pos (Deact)							
N/A							

*Note*: Minimum *t*‐stat threshold = 3.14. All results obtained using an uncorrected voxel‐wise threshold of *p* < .001 and a FDR corrected threshold of *p* < .05.

Abbreviations: Act, activation; Deact, deactivation; dmPFC, dorsomedial prefrontal cortex; FDR, false discovery rate; MNI, Montreal Neurological Institute.

#### Direct contrasts

Examining the findings of the direct contrasts, when children experienced negative movie time‐series fluctuations compared to positive time‐series, the HBQ Overanxious subscale was significantly positively associated with elevated activation in the dmPFC (*t* = 4.26, *p* < .001), right MTG (*t* = 3.66, *p* = .001), and left thalamus (*t* = 3.42, *p* = .013).

### ICA analyses

#### Baseline analysis

Temporal sorting identified 15 task‐related components significantly associated with the movie‐content fluctuation regressors at *p* < .001, uncorrected (Calhoun et al., 2008; Malinen & Hari, 2011). Across these components, the temporal sorting model explained substantial variance in component time courses (*R* ^2^ = .84). Of these components, 9 were preferentially associated with negatively valenced fluctuations, whereas 6 were preferentially associated with positively valenced fluctuations.

### MANCOVAN analysis

**FIGURE 1:**
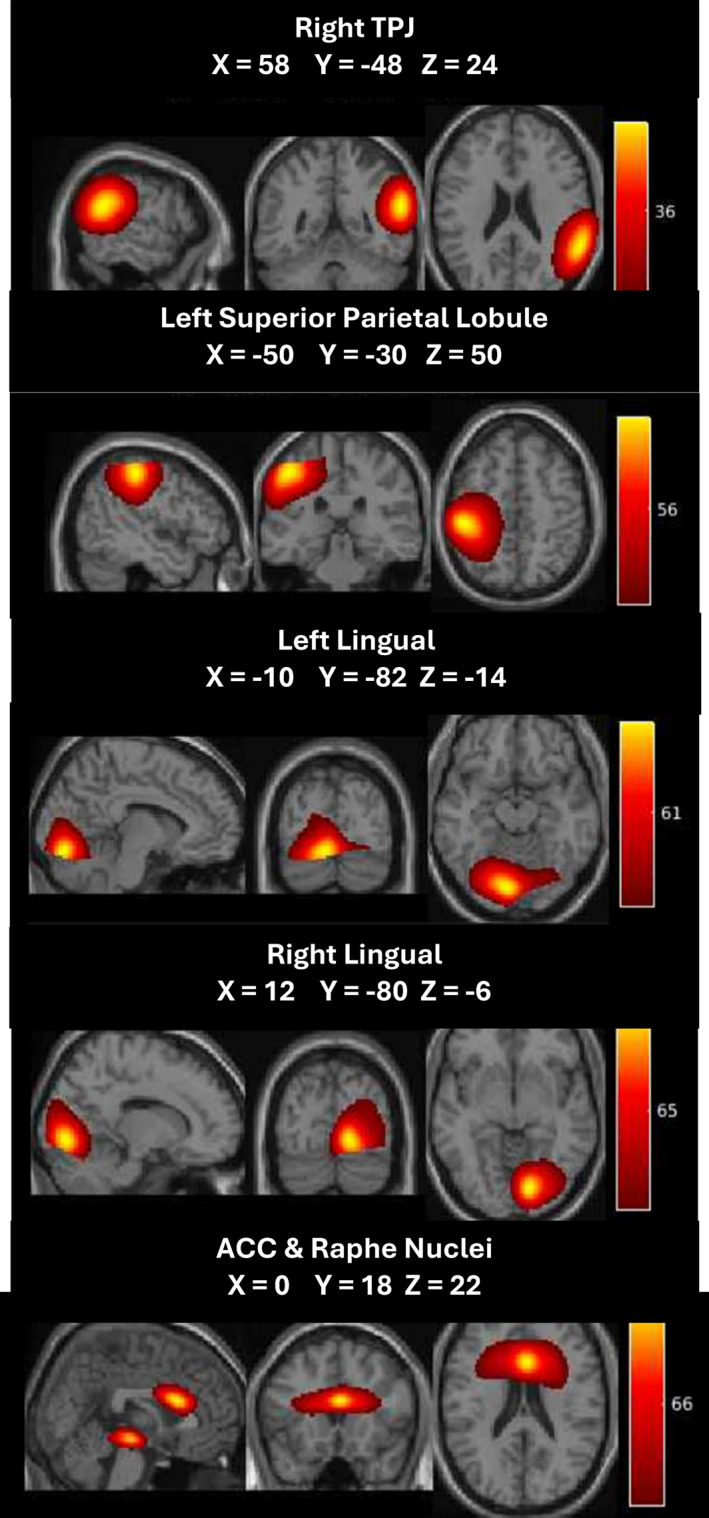
Composite orthogonal viewpoints of the five spatial components that significantly covary with the Health and Behavior Questionnaire Overanxious subscale (estimated through the MANCOVAN application of the GIFT Toolbox; van der Meer et al., 2020).

**FIGURE 2:**
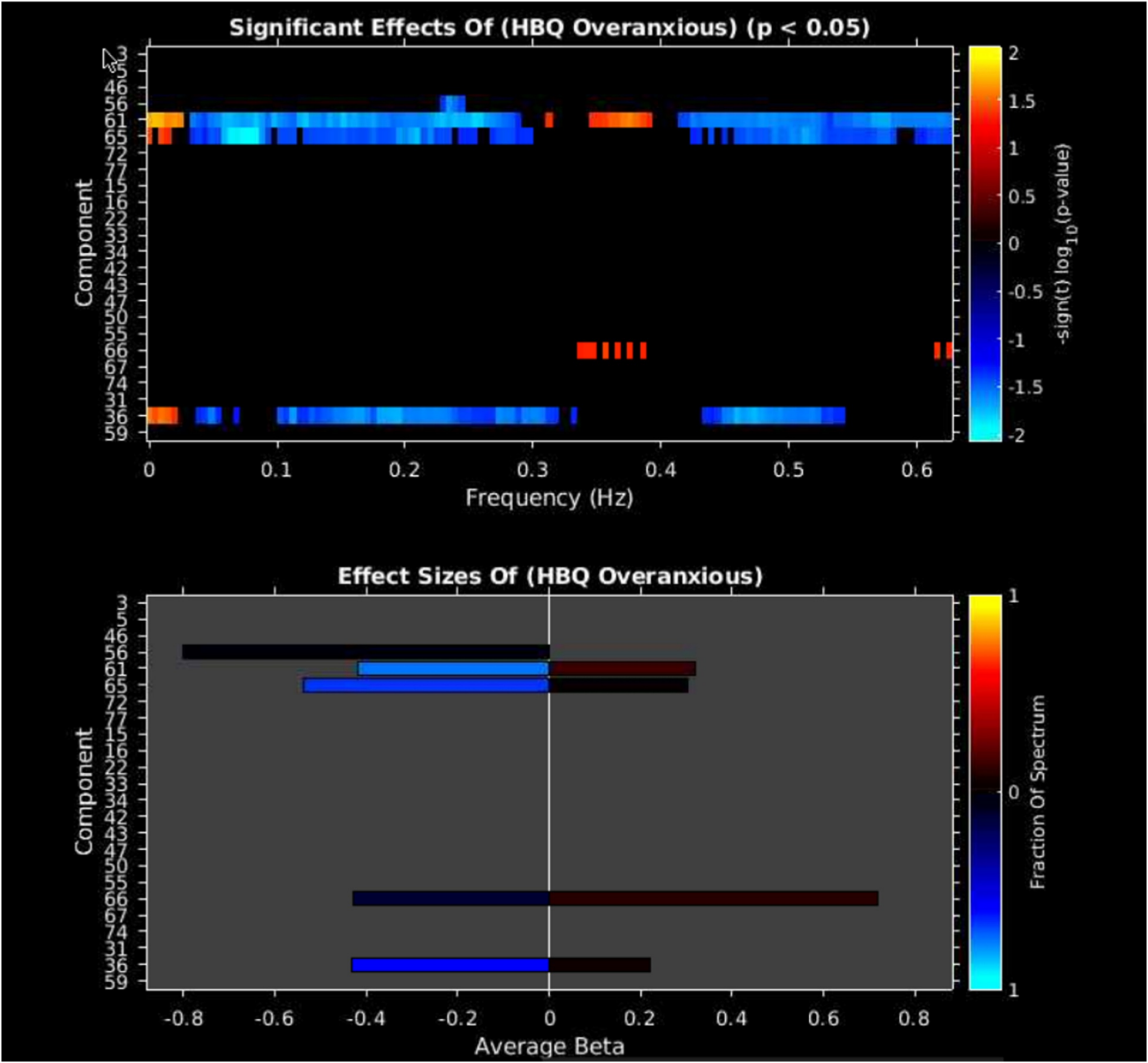
Univariate results of associations between HBQ Overanxious subscale and component power spectra generated from the MANCOVAN package launched through GIFT toolbox. Associations are visualized by plotting the log of the *p*‐value with the sign of the associated *t*‐statistic, −sign (*t*) log_10_ (*p*). Effect sizes are plotted as the average standardized beta coefficients of the association between the component power spectra and HBQ Overanxious. The color of the bar indicates the fraction of the component contributing to the positive or negative effect. HBQ, Health and Behavior Questionnaire.

All components displaying significant associations with the HBQ Overanxious subscale are illustrated in Figure 1. Greater anxious symptoms were associated with substantially diminished spectral power spanning lower‐ and higher‐frequency ranges in the right temporoparietal junction (TPJ; component 36) and lingual gyri (components 61 and 65) when exposed to negative fluctuations in the video time‐series. Furthermore, greater anxious symptoms were associated with elevated high frequency power in the ACC and the raphe nuclei (component 66). Finally, higher anxious symptoms were associated with diminished high frequency power in the left parietal lobule (component 56). The fraction of the frequency spectrum in the right TPJ and lingual gyri components contributing to the negative medium effect in the low‐frequency band was substantially higher than the fraction contributing to the positive small effect in the same bandwidth. In contrast, the ACC and raphe nuclei exhibited a larger overall effect size (weighted *β* = .73) relative to the right TPJ (weighted *β* = −.43) and lingual gyri (IC61: weighted *β* = −.42; IC65: weighted *β* = −.54), despite contributing a smaller fraction of the spectrum. While the left superior parietal lobule showed a comparably large effect size (weighted *β* = −.80), the fraction of the spectrum contributing to this effect was minimal (3.1%). Power spectra effects surviving FDR correction (*p*_fdr_ < .05) are displayed in Figure 2, with component‐level and cluster‐level statistics reported in Supporting Information S1: Tables S1 and S2.

ACE total score were significantly correlated with HBQ Overanxious score (*r* = .26, *p* = .001), supporting the relevance of ACE exposure when interpreting anxiety‐related neural findings. Supplementary ACE‐adjusted analyses showed that the strongest HBQ Overanxious‐related spectral effects remained in the right TPJ (IC36) and right lingual gyrus (IC65) after accounting for ACE exposure (Supporting Information S1: Figure S1; Tables S3 and S4). Specifically, higher HBQ Overanxious scores remained associated with lower spectral power across 44.2% of the spectrum in the right TPJ (weighted *β* = −.47) and 37.2% of the spectrum in the right lingual gyrus (weighted *β* = −.51). Effects previously observed in the left superior parietal lobule (IC56), left lingual gyrus (IC61), and ACC/raphe nuclei (IC66) did not survive the ACE‐adjusted sensitivity analysis.

**FIGURE 3:**
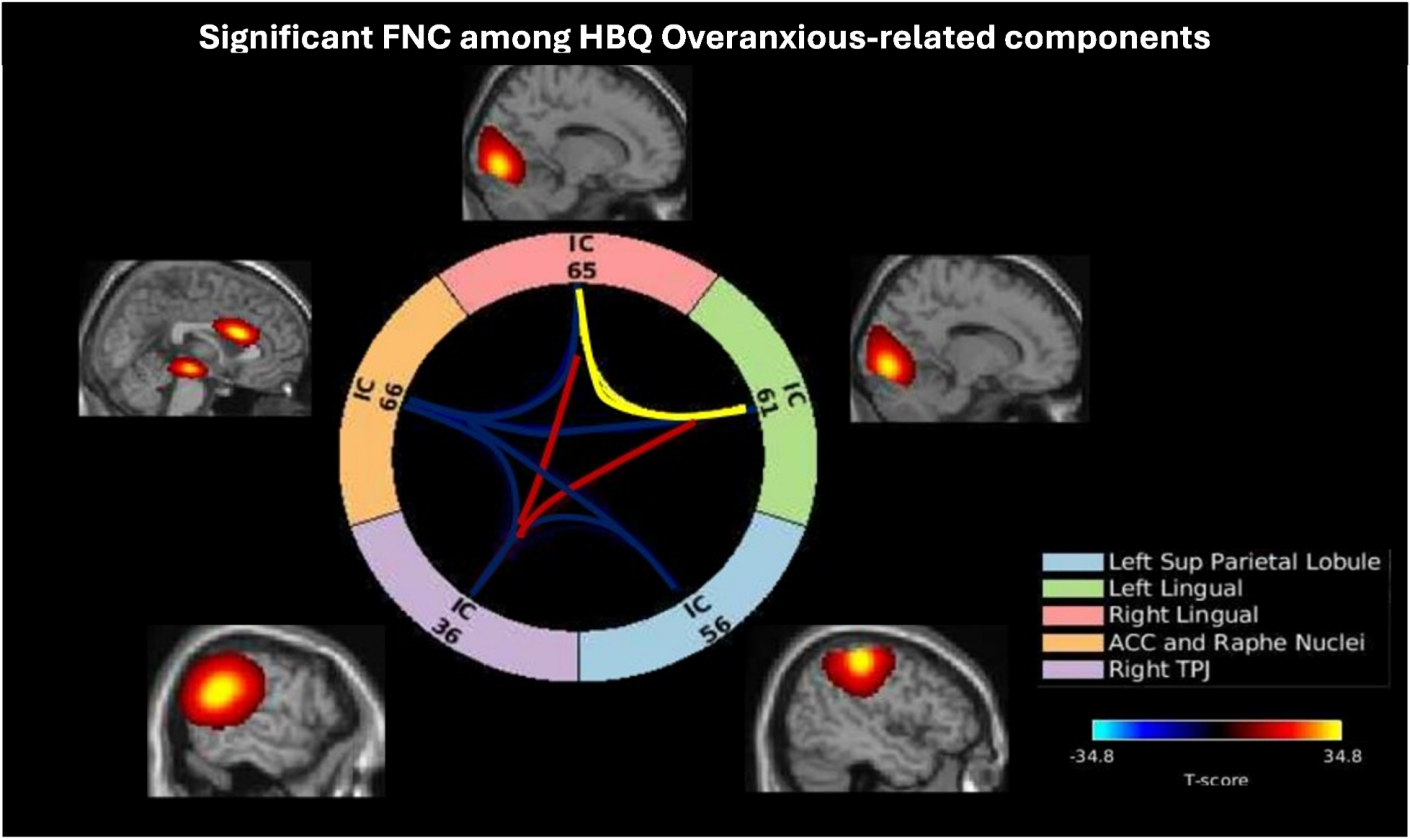
A connectogram displaying group‐level FNC among HBQ Overanxious‐related components. Connection strength is quantified by *t*‐statistics derived from one‐sample *t*‐tests on Fisher‐*z* transformed correlations. All reported connections survived FDR correction (*p*_fdr_ < .001). FDR, false discovery rate; FNC, functional network connectivity; HBQ, Health and Behavior Questionnaire.

Finally, significant group‐level FNC among the HBQ Overanxious‐related components identified above was observed (Figure 3). Specifically, strong positive connectivity was detected between the left and right lingual gyri (IC61–IC65; *t* = 29.13, *p*_fdr_ < .001). In contrast, the ACC and raphe nuclei (IC66) exhibited significant negative connectivity with the right TPJ (IC36–IC66: *t* = −4.49, *p*_fdr_ < .001), the left lingual gyrus (IC61–IC66: *t* = −8.77, *p*_fdr_ < .001), and the left superior parietal lobule (IC56–IC66: *t* = −6.47, *p*_fdr_ < .001). Positive connectivity was additionally observed between the right TPJ and both lingual gyri (IC36–IC61: *t* = 4.42, *p*_fdr_ < .001; IC36–IC65: *t* = 5.24, *p*_fdr_ < .001).

## DISCUSSION

The objective of the current study was to investigate children's dynamic brain response to anticipatory fear as a function of their anxiety symptoms in early childhood. We found that neural fluctuations in the dmPFC, right MTG, and left thalamus during the negatively‐valenced time series were positively associated with anxiety symptoms. Furthermore, during the negatively‐valenced time series, diminished connectivity between the (1) ACC and raphe nuclei and the (2) the right TPJ, left superior parietal lobule, and lingual gyrus was detected. Heightened signal power was also identified in the ACC and raphe nuclei while power was diminished in the right TPJ, left superior parietal lobule and lingual gyri.

Prior to broad discussion of our findings, we acknowledge the limitations of our study. First, although we simulated a naturalistic context typical of a child's daily entertainment viewing, the constraints of the MRI environment limit ecological validity in social contexts. There may also be a disconnect in how individuals process anticipatory fear while viewing entertainment compared to a personal threatening experience. Future research could increase the ecological validity of free‐viewing paradigms by utilizing alternative modalities (e.g., fNIRS, mobile eye‐tracking) that would allow for interactive engagement with real‐world threat. Second, while parents reported on the anxiety symptoms that they observe in their child, a clinical interview was not completed and there are likely low rates of meeting diagnostic anxiety criteria in our sample, which is expected in a community recruited sample of this age. Nevertheless, in early childhood, temperament and early symptoms are identified risk factors for later anxiety disorder diagnosis (Hudson et al., 2019; Mesman & Koot, 2001). Third, while our study deeply phenotyped neural response to anticipatory fear through moment‐to‐moment fluctuations in brain activation, it lacked further biological and behavioral characterization. Future studies could use methods that are less sensitive to movement (e.g., psychophysiology, eye‐tracking) in combination with observed behavior in a child's naturalistic environment, which may help to collect the comprehensive profiles necessary to best detect children who are most at risk for anxiety diagnosis. Fourth, ACE‐related sensitivity analyses attenuated the broader pattern of findings, though the strongest anxiety‐related spectral effects remained evident in the right TPJ and right lingual gyrus. Nevertheless, the adversity context of this sample calls for interpretive caution. ACE exposure was significantly correlated with HBQ Overanxious scores (*r* = .26, *p* = .001), and both ACE burden and interparental conflict have been linked to childhood anxiety symptoms and altered neural threat‐processing (Bounoua et al., 2022; Elmore & Crouch, 2020; Lopez‐Tamayo et al., 2022; Ran et al., 2021). How these factors interact with one another and with the neural mechanisms underlying early anxiety is a question that longitudinal designs may be better positioned to answer. Finally, from a computational perspective, although Pearson correlations can capture stable FNC during movie viewing, this static approach simplifies the dynamic nature of brain connectivity by measuring only zero‐lag linear associations. Future studies should employ dynamic connectivity methods to better characterize temporal fluctuations in network interactions during naturalistic viewing.

While our neural pattern of regional findings did not map directly onto the larger body of findings in children with anxiety, our study does replicate the regional results of Geng et al. (2018). In adults with high trait anxiety subjects exhibited increased activation in the dmPFC, MTG, and thalamus during the anticipation of uncertain threats using a traditional experimental paradigm presenting static emotional images. Altered mPFC network function has been identified as sign of heightened anxiety risk in late childhood and adolescence, with evidence of dysregulated activity and connectivity within and between the PFC and other structures (Birn et al., 2014; Clauss et al., 2016; Strawn et al., 2014). Furthermore, research on children with a predisposition to anxiety has shown that abnormal connectivity between the MTG and other brain regions, particularly in response to social and emotional stimuli, may be linked to the later development of anxiety symptoms (Makovac et al., 2016). The maturation of thalamic‐prefrontal connectivity during childhood and adolescence also plays a crucial role in regulating anticipatory responses (Mair et al., 2022). Children with elevated anxiety symptoms may already exhibit heightened engagement of neural systems involved in monitoring uncertainty, interpreting emotionally salient social information, and preparing for potential threat. The dmPFC is broadly implicated in evaluative processing, self‐referential cognition, and the monitoring of ambiguous or uncertain socioemotional information (Carleton, 2016; Grupe & Nitschke, 2013). Its heightened engagement during negatively‐valenced fluctuations may therefore reflect a tendency in anxious children to over‐monitor or over‐interpret potential threat, even within mildly threatening, developmentally typical contexts (Geng et al., 2018). The MTG may support the integration of social and narrative information during naturalistic viewing (Pantelis et al., 2015; Vanderwal et al., 2019), suggesting that elevated anxiety symptoms are associated with altered processing of emotionally salient story content. The thalamus, as a hub for sensory gating and cortico‐subcortical communication, may further amplify the salience of negative or uncertain cues (Geng et al., 2018; Mair et al., 2022). Taken together, heightened engagement of this dmPFC–MTG–thalamus circuit suggests that early anxiety symptoms may arise not only from exaggerated fear responses to explicit threats, but also from an increased neural sensitivity to dynamic, ambiguous, and socially embedded cues that signal potential danger. This pattern may help explain why anxiety symptoms emerge with peak prevalence as early as age 5.5 (Uhlhaas et al., 2023). Thus, it is possible that engagement of this neural circuitry might serve as a salient and detectable risk indicator for future anxiety diagnosis as children develop; however, longitudinal studies would be needed as children age to the middle childhood and adolescent period in which anxiety disorders are typically diagnosed (Kessler et al., 2012). Notably, for children in the current sample who were additionally exposed to interparental conflict and household stress, repeated experience of an unpredictable and emotionally charged home environment may further sensitize these neural systems to anticipatory threat, amplifying the patterns observed here. It should be acknowledged that the present findings remain contingent on adult‐derived emotion ratings of the stimulus material rather than on emotional responses obtained directly from children. Novel, age‐appropriate approaches, such as simplified categorical rating scales, passive physiological monitoring, or eye‐tracking, will be valuable for capturing children's dynamic emotional responses more directly and further strengthening the ecological validity of future neuroimaging studies in this age group.

Moreover, our results uncovered evidence of abnormal power spectra and network connectivity among the right TPJ, ACC, raphe nuclei, left superior parietal lobule and lingual gyri in association with anxiety symptoms. Heightened anxiety symptoms were associated with exaggerated high frequency power oscillations in the ACC and raphe nuclei, regions implicated in various facets of emotional response and mood regulation in children and adults (Horn and ung, 2012; Stevens et al., 2011). These regions are notably active when individuals must regulate emotional responses to social, especially threat‐oriented stimuli (Balázsfi et al., 2018; Rotge et al., 2015). However, pervasive high frequency bandwidths are typically indicative of dysfunction in signal propagation from local to distal networks meant to facilitate adaptive functioning in the individual (Cordes et al., 2000). This may indicate a continuous perturbed state in the children in our sample with the most anxious symptoms. Furthermore, elevated anxiety symptoms were associated with diminished low frequency power in the right TPJ and lingual gyri, regions frequently recruited in tasks requiring social cognition, perception, and language processing (Ahmad et al., 2021; Deuse et al., 2016). This may suggest that children with high anxious symptomotology had difficulties in processing the intentions and behavior of the characters during negative moments in the show, compromising their ability to effectively regulate the emotional content filtered through the ACC and raphe nuclei. This impairment is further underscored by the results of connectivity analyses, which revealed a broad disconnection between the ACC and raphe nuclei and the right TPJ and lingual gyri. In adults with social anxiety disorder, the ACC, TPJ, and raphe nucleus have been implicated in comprising a neurobehavioral network profile of the disorder, one characterized by heightened emotional reactivity and impairments in perspective‐taking. Diminished connectivity in this network might leave anxious children predisposed to the inaccurate decoding of emotional information which precipitate inappropriate or maladaptive responses during times of uncertainty, especially in response to threat. Recent complementary research in the field of naturalistic audiovisual‐based neuroimaging research has employed alternative methods such as hidden Markov modeling to detect latent features of unstructured or nonlinear imaging data (Sava‐Segal et al., 2023). One study in a larger age range including older children also found that time spent in unique brain states during movie viewing correlated with anxiety symptoms (Camacho et al., 2025).

In summary, our study is aligned with a recent model suggesting the presence of sensitive periods for the salient expression of anxiety (Uhlhaas et al., 2023) by investigating fluctuations in neural activation during anticipatory fear as a function of anxiety symptoms in early childhood. The findings of this study can serve as a foundation in the identification of psychiatric symptoms before the traditionally defined onset and makes early strides in translating the theoretical frameworks that have been delineated in the preschool literature (Egger & Angold, 2004; Luby et al., 2014; Wakschlag et al., 2018). Our findings can potentially be translated into understanding mechanistic brain‐behavior relationships that are paramount to generating holistic improvements in treatment approaches for children with early anxious symptoms. The differential neural signatures identified in this study may serve as a precursor to the development and maintenance of anxiety in adulthood.

## AUTHOR CONTRIBUTIONS

**Khalil I. Thompson**: Methodology; data analysis; validation; visualizations; writing—original draft; writing—review and editing. **Yiwen Zhang**: Validation; writing—original draft; writing—review and editing. **Denny Schaedig**: Methodology; data curation; data analysis; writing—review and editing. **Anna Bankston**: Data collection; data analysis; writing—original draft; writing—review and editing. **Anoushka Parnerkar**: Data collection; writing—original draft; writing—review and editing. **Shrinidhi Jeyaram**: Data collection; data analysis. **Nadine Melhem**: Conceptualization; methodology; writing—review and editing; project administration; resource management; funding acquisition. **Susan B. Perlman**: Conceptualization; methodology; writing—review and editing; supervision; project administration; resource management; funding acquisition.

## CONFLICT OF INTEREST STATEMENT

The authors declare no conflicts of interest.

## ETHICAL CONSIDERATIONS

This study was approved by the Institutional Review Board at Washington University in St. Louis on July 28, 2020 (IRB ID: 202007029). All parents provided written informed consent, and children provided verbal assent.

## AI STATEMENT

The authors declare that they have not used AI in any capacity that would require disclosure in accordance with Wiley's AI Guidelines, nor in any capacity that would reasonably require disclosure for editors, reviewers, and readers to properly evaluate their research or manuscript. The authors confirm that AI has not been used for purposes including, but not limited to, drafting and editing, generating substantial text or restructuring arguments, or in the research methodology. The authors take full responsibility for the accuracy of this statement.

## Supporting information

Supporting Information S1

## Data Availability

The data that is the subject of this article is publicly available through the National Data Archive.
