# Supplementary material for: Identifiable regional and neural network abnormalities in early childhood anxiety
Source: JCPP Adv. 2026 Aug 16:e70158. Online ahead of print. doi: 10.1002/jcv2.70158 (PMC13478735; doi:10.1002/jcv2.70158)
Supplement: Supplementary file 1 [file JCV2-9999-e70158-s001.docx]

**Identifiable Regional and Neural Network Abnormalities in Early Childhood Anxiety**

**Supporting Information**

**Figure S1.** Univariate results were generated using the MANCOVAN package in the GIFT toolbox after including ACE exposure as an additional covariate. Significant associations between HBQ Overanxious scores and component power spectra are visualized by plotting the signed log-transformed p-value, −sign(t)log10(p). Effect sizes are plotted as the average standardized beta coefficients for the association between component power spectra and HBQ Overanxious scores after accounting for ACE exposure. The color of each bar indicates the fraction of the component spectrum contributing to the positive or negative effect. After ACE adjustment, significant spectral effects remained evident primarily in the right TPJ (IC36) and right lingual gyrus (IC65).

**Table S1. Component-level summary of associations between HBQ Overanxious and spectral power**

| **Name of Region (Component)** | **Direction** | **Total Fraction of Spectrum (%)** | **Effect Size (Weighted *β*)** |
| --- | --- | --- | --- |
| Right TPJ (IC 36) | Positive | 3.9% | 0.22 |
|  | Negative | 57.4% | -0.43 |
| Left superior parietal lobule (IC56) | Negative | 3.1% | -0.80 |
| Left Lingual gyrus (IC61) | Positive | 13.2% | 0.32 |
|  | Negative | 75.2% | -0.42 |
| Right Lingual gyrus (IC65) | Positive | 2.3% | 0.30 |
|  | Negative | 66.7% | -0.54 |
| ACC and raphe nuclei (IC66) | Positive | 7.0% | 0.73 |

*Note:* Effect sizes are presented as average standardized beta coefficients across significant frequency bins. Fraction of spectrum indicates the proportion of the frequency bandwidth contributing to each effect.

**Table S2 Cluster-level test statistics for spectral power changes associated with HBQ Overanxious**

| **Region / Component** | **Direction** | **Frequency range (Hz)** | **Fraction of spectrum (%)** | **Cluster *β*** | **Peak t-value** | **FDR-p value** |
| --- | --- | --- | --- | --- | --- | --- |
| Right TPJ (IC36) | Negative | 0.103–0.317 | 34.9 | -0.44 | -2.99 | 0.035 |
|  | Negative | 0.435–0.542 | 17.8 | -0.46 | -2.55 | 0.021 |
| Left superior parietal lobule (IC56) | Negative | 0.229–0.244 | 3.1 | -0.8 | -3.53 | 0.022 |
| Left Lingual gyrus (IC61) | Positive | 0.000–0.024 | 4.7 | 0.37 | 2.21 | 0.019 |
|  | Negative | 0.034–0.288 | 41.1 | -0.44 | -3.14 | 0.017 |
| Right Lingual gyrus (IC65) | Negative | 0.034–0.093 | 10.1 | -0.47 | -3.65 | 0.012 |
|  | Negative | 0.103–0.112 | 2.3 | -0.4 | -2.06 | 0.036 |
|  | Negative | 0.122–0.234 | 18.6 | -0.53 | -2.92 | 0.020 |
|  | Negative | 0.269–0.298 | 5.4 | -0.53 | -2.05 | 0.036 |
|  | Negative | 0.459–0.581 | 20.2 | -0.62 | -2.87 | 0.020 |
|  | Negative | 0.601–0.625 | 4.7 | -0.49 | -1.97 | 0.038 |
| ACC and raphe nuclei (IC66) | Positive | 0.337–0.347 | 2.3 | 0.69 | 2.61 | 0.046 |

*Note:* Significant frequency clusters were identified using an FDR-corrected threshold of *p* < .05. To highlight the most robust findings, the table reports dominant clusters with a peak |t| > 1.96 that contribute at least 2% to the total frequency spectrum. Cluster *β* represents the average standardized *β* coefficient across significant bins within the cluster. Peak t-statistics represent the maximum absolute t-value within the cluster boundaries

**Table S3. Component-level summary of associations between HBQ Overanxious and spectral power controlling the effects of ACE**

| **Name of Region (Component)** | **Direction** | **Total Fraction of Spectrum (%)** | **Effect Size (Weighted β)** |
| --- | --- | --- | --- |
| Right TPJ (IC36) | Positive | 3.1% | 0.23 |
|  | Negative | 44.2% | -0.47 |
| Right Lingual gyrus (IC65) | Positive | 0.8% | 0.30 |
|  | Negative | 37.2% | -0.51 |

*Note:* This sensitivity analysis included ACE exposure as an additional covariate. Effect sizes are presented as average standardized beta coefficients across significant frequency bins. Fraction of spectrum indicates the proportion of the frequency bandwidth contributing to each effect. After accounting for ACE exposure, significant HBQ Overanxious-related spectral effects remained for the right TPJ (IC36) and right lingual gyrus (IC65), whereas effects in IC56, IC61, and IC66 were no longer detected.

**Table S4 Cluster-level test statistics for spectral power changes associated with HBQ Overanxious controlling the effects of ACE**

| **Region / Component** | **Direction** | **Frequency range (Hz)** | **Fraction of spectrum (%)** | **Cluster *β*** | **Peak t-value** | **FDR-p value** |
| --- | --- | --- | --- | --- | --- | --- |
| Right TPJ (IC36) | Negative | 0.122–0.239 | 19.4 | -0.47 | -2.95 | 0.023 |
|  | Negative | 0.273–0.317 | 7.8 | -0.46 | -2.11 | 0.026 |
|  | Negative | 0.444–0.522 | 13.2 | -0.53 | -2.63 | 0.022 |
| Right Lingual gyrus (IC65) | Negative | 0.049–0.093 | 7.8 | -0.48 | -3.48 | 0.018 |
|  | Negative | 0.137–0.151 | 3.1 | -0.47 | -2.20 | 0.047 |
|  | Negative | 0.161–0.195 | 6.2 | -0.49 | -2.48 | 0.046 |
|  | Negative | 0.205–0.215 | 2.3 | -0.57 | -2.59 | 0.046 |

*Note:* Significant frequency clusters were identified using an FDR-corrected threshold of *p* < .05 after including ACE exposure as an additional covariate. To highlight the most robust ACE-adjusted findings, the table reports dominant clusters with a peak |t| > 1.96 that contribute at least 2% to the total frequency spectrum. Cluster *β* represents the average standardized *β* coefficient across significant bins within the cluster. Peak t-statistics represent the maximum absolute t-value within the cluster boundaries. FDR-*p* values indicate the minimum q-value within the cluster.
